# Preparation, Characterization, and Bioactivity Evaluation of Polyoxymethylene Copolymer/Nanohydroxyapatite-g-Poly(ε-caprolactone) Composites

**DOI:** 10.3390/nano12050858

**Published:** 2022-03-03

**Authors:** Kinga Pielichowska, Paula Szuba, Joanna Maciocha, Beata Macherzyńska, Katarzyna Nowicka, Piotr Szatkowski

**Affiliations:** Department of Biomaterials and Composites, Faculty of Materials Science and Ceramics, AGH University of Science and Technology, Al. Mickiewicza 30, 30-059 Kraków, Poland; paula.szuba@wp.pl (P.S.); joannamaciocha93@gmail.com (J.M.); beatam@agh.edu.pl (B.M.); nowicka@agh.edu.pl (K.N.); pszatko@agh.edu.pl (P.S.)

**Keywords:** polyoxymethylene, functionalization, nanohydroxyapatite, thermal properties, mechanical properties

## Abstract

In this work, nanohydroxyapatite (HAp) was functionalized with poly(ε-caprolactone) (PCL), using 1,6-hexamethylene diisocyanate (HDI) as a coupling agent, and then incorporated into the polyoxymethylene copolymer (POM) matrix using the extrusion technique. The obtained POM/HAp-g-PCL composites were investigated using FTIR, DSC, TOPEM DSC, and TG methods. Mechanical properties were studied using destructive and non-destructive ultrasonic methods, wettability, and POM crystallization kinetics in the presence of HAp-g-PCL. Moreover, preliminary bioactivity evaluation of the POM/HAp-g-PCL composites was performed using the Kokubo method. It was found that the introduction of HAp-g-PCL to the POM matrix has a limited effect on the phase transitions of POM as well as on its degree of crystallinity. Importantly, HAp grafted with PCL caused a significant increase in the thermal stability of the POM, from 292 °C for pristine POM to 333 °C for POM modified with 2.5% HAp-g-PCL. If unmodified HAp was used, a distinct decrease in the thermal stability of the POM was observed. Crystallization kinetic studies confirmed that HAp-g-PCL, in small amounts, can act as a nucleating agent for the POM crystallization process. Moreover, incorporation of HAp-g-PCL, although slightly decreasing the mechanical properties of POM composites, improved the crucial parameter in biomedical applications, namely the in vitro bioactivity.

## 1. Introduction

Polyoxymethylene (POM) is a well-known thermoplastic engineering resin that exhibits good mechanical properties, stiffness, creep and fatigue resistance, moldability, and excellent chemical resistance [1]. POM is widely used in many different applications, such as automotive applications, some parts of precision machines, and electrical and electronic applications, as well as in biomedical applications [2,3]. Due to its excellent mechanical properties in the latter field, POM can be applied in bone tissue replacement [4].

To make POM more biocompatible and bioactive in bone tissue engineering applications, it can be modified with hydroxyapatite (HAp), which is the compound most similar to the mineral part of human bonds [5,6]. However, in our previous work, it has been revealed that the incorporation of higher amounts of HAp into the POM matrix leads to a significant decrease in POM thermal stability. It was also found that HAp acts as a catalyst for the POM depolymerization reaction by decreasing the activation energy of POM thermal degradation [7]. We also found that HAp functionalization by poly(ethylene glycol) (PEG) grafting leads—through blocking of POM basic active sites—to significant improvements in the thermal stability of polyoxymethylene [8,9].

In HAp single crystal lattice, there are both acidic and basic active sites present; at a Ca/P ratio of 1.50, HAp acts more as an acid catalyst, while at a stoichiometric Ca/P ratio of 1.67 it behaves like a basic catalyst. On the other hand, when Ca/P ratio is between 1.50 and 1.67, HAp can be characterized by both an acid and a basic character [10,11,12]. Generally, the bond between oxygen and carbon atoms in oxymethylene units is very sensitive to different factors, such as high temperature or the presence of acids or alkali. The main chain of the POM copolymer consists of oxymethylene mers with a small number of randomly distributed oxyethylene units. The incorporation of oxyethylene mers, which are more thermally stable, enhances the thermal stability of POM. The scission of C-O bonds in oxymethylene units results in the depolymerisation of the POM main chain with formaldehyde releasing. Formaldehyde can undergo oxidation to formic acid, and both formaldehyde and formic acid also accelerate POM chain degradation through depolymerization [13].

In our work, we aimed at the incorporation of higher amounts of HAp into the POM matrix while avoiding the unfavorable effect of HAp on the thermal stability of the polymer, and the proposed solution is the functionalization of HAp with poly(ε-caprolactone) diol (PCL) (HAp-g-PCL), using 1,6-hexamethylene diisocyanate as a coupling agent, to block the basic active sites on the HAp surface. After HAp functionalization, HAp-g-PCL inorganic–organic hybrid filler was incorporated into the POM matrix using melt processing methods. In this work, the effect of novel hybrid HAp-g-PCL filler on the selected properties of POM/HAp-g-PCL properties such as thermal behavior, melting, crystallization kinetics, mechanical properties, and in vitro chemical stability has been investigated.

## 2. Materials and Methods

Commercially available POM copolymer (Ultraform^®^, BASF, Ludwigshafen, Germany) with melt flow rate (MFR (190/2.16, ISO 1133) 2.6 g/10 min has been used for composite preparation. The average molar mass was determined using the GPC method according to ASTM D5296-05 at a temperature 40 °C with the VE2001 Viscotek GPC system equipped with the RI-VE 3580 Viscotek detector (PL HFIPgel, 605 E901506 and HFIP 603 E910606 columns). Average molar mass has been determined based on the polystyrene standard as: *M_n_* = 8 269, *M_w_* = 78 565 and dispersity *M_w_*/*M_n_* = 8.96. Stoichiometric spherical-shaped hydroxyapatite (HAp) with general chemical formula C_10_(PO_4_)_6_(OH)_2_ was supplied from nGimat Co. (Atlanta, USA). The diameter of 99% HAp particles was below 100 nm. 1,6-heksamethylenodiisocyanate (HDI), poly(ε-caprolactone) diol (PCL) with average molar mass of 2000 g/mol and dibutyltin dilaurate (DBTDL) were supplied from Sigma Aldrich and used as received. Anhydrous *N*,*N*-dimethylformamide (DMF) and ethanol were produced by Avantor (Gliwice, Poland).

### 2.1. HAp Functionalization and POM/HAp-g-PCL Composites Preparation

**Figure 1:**
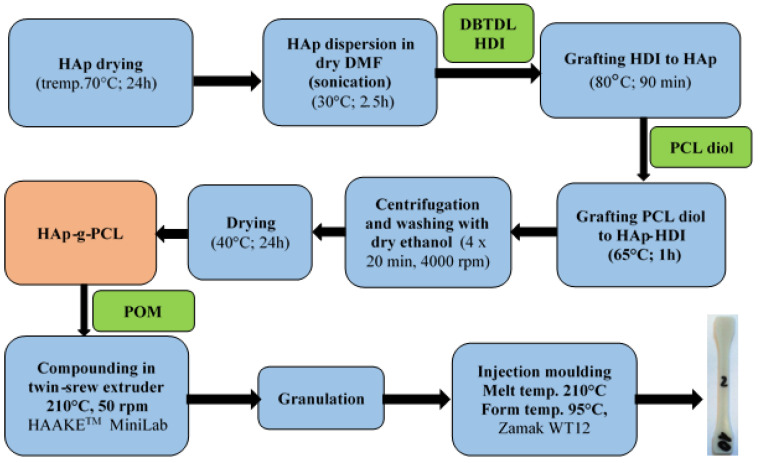
Preparation of POM/HAp-g-PCL composites.

HAp has been functionalized according to the procedure described in [8,9], but instead of PEG, PCL diol with an average molar mass of 2000 g/mol has been used. The molar ratio HAp:HDI:PCL was 1:2:2. The general protocol for the preparation of POM/HAp-g-PCL composites is presented in Figure 1.

First, HAp was dried for 24 h at 70 °C. Then, HAp was introduced to a three-necked round bottom flask with 90 mL of anhydrous DMF and dispersed by sonication under dry nitrogen. After sonication, 9 μL of DBTDL catalyst was introduced to the obtained dispersion and mixed using a mechanical stirrer. Solution of 6 mL HDI in 12 mL DMF was dropped to HAp dispersion and temperature was increased to 80 °C and kept for 1.5 h. The reaction mixture was then cooled to 40 °C and a 36 g PCL solution in 36 mL of DMF was added to the reaction mixture. The temperature was increased to 65 °C and the mixture was stirred for 1 h under nitrogen. After synthesis, the powder was separated by centrifugation (4000 rpm for 20 min), washed three times with ethanol and, after each washing, separated by centrifugation. The sediment obtained (HAp-g-PEG) was dried for 24 h at 40 °C.

The obtained HAp-g-PCL was compounded with POM using melt processing methods. Based on TGA results for HAp-g-PCL, it has been introduced to the POM matrix in the appropriate amounts to obtain composites with 0.5%, 1.0%, 2.5%, 5.0%, and 10% of HAp (after recalculation to unmodified HAp). In the first step, HAp-g-PCL was compounded with POM using a HAAKE TM MiniLab twin screw extruder at 210 °C and 50 rpm. The POM/HAp-g-PCL filament obtained was granulated and then shaped by injection molding using a Zamak WT12 injection molder. The melt temperature was 210 °C, while the mold temperature was 95 °C. Unmodified POM has also been melt processed using the same procedure to obtain reference samples with the same thermal history.

### 2.2. Techniques

FTIR measurements have been performed using a Brucker Tensor 27 spectrometer, for HAp-g-PCL powder in KBr pellets, and for POM/HAp-g-PCL using FTIR-ATR method on diamond crystal. Measurements were carried out in the range 400–4000 cm^−1^ with a resolution of 2 cm^−1^. For DSC investigations, the differential scanning calorimeter DSC1 from Mettler Toledo with intracooler cooling has been used in both dynamic and isothermal options, as well as in TOPEM mode. In dynamic option samples, ca. 5.7 mg were placed in aluminum pierced pans and heated/cooled with underlying rate of 10 K/min in the temperature range −10 °C–200 °C in nitrogen atmosphere (30 mL/min). For isothermal crystallization kinetic studies, samples were heated to 200 °C, held isothermally for 5 min at 200 °C, then rapidly cooled with cooling rate 80 °C/min to isothermal crystallization temperature, held isothermally at selected temperature to the end of crystallization, and finally heated up again to 200 °C to determine the melting temperature after isothermal crystallization at selected temperatures. Isothermal crystallization was performed at four different temperatures. TOPEM DSC measurements were carried out with an underlying heating rate of 2 K/min, an amplitude ±0.5 K and a switching time of 15–50 s.

Thermogravimetric analysis (TGA) was performed using the TGA 550 Discovery thermal analyzer from TA Instruments. Samples with mass ca. 7 mg were placed in platinum pans and heated with a heating rate of 10 K/min in the temperature range 40–600 °C under nitrogen atmosphere (20 mL/min).

The densities (*D_s_*) of the samples were determined using hydrostatic weighing method at room temperature and calculated using Equation (1).

(1) $$D_{ps}=\frac{m_{s}}{m_{n}-m_{w}}\cdot d_{o}$$

where *m_s_* is the sample mass in air (g); *m_w_* is the sample mass in water (g); *m_n_* is the mass of wet sample in air (g); and *d_o_* is the water density at measurement temperature (g/cm^3^).

Young modulus and tensile strength were determined using Zwick 1435 tensile testing machine according to the PN-EN ISO 527. Injection-molded dumbbell specimens shaped according to ISO 3167 with thickness 1.92 ± 0.07 mm and width 4.78 ± 0.08 mm were applied. The mechanical properties presented were calculated by averaging measurements for three specimens.

For ultrasonic measurements, the ultrasonic measuring system UZP-1 (INCO-VERITAS) was employed. An ultrasonic pulse technique was used to determine the elastic properties (*E*). The 10 MHz transducer with a diameter of 10 mm connected to a sample by paraffin oil was used for longitudinal waves, and the same transducer was glued with the samples by the Canadian balm for transverse waves. The values of the material constants were calculated using the following formula [14] Equation (2).

(2) $$E=D_{P}\cdot {\left(C_{L}\right)}^{2}\cdot \left(1-\nu^{2}\right)$$

where *E* is ultrasonic Young’s modulus, *ν* is Poisson’s Ratio, *D_p_* is apparent density, and *C_L_* is the speed of a longitudinal wave. Measurements were performed on three samples, and standard deviation was calculated and presented.

Wettability was determined by measuring contact angles using a drop shape analysis system DSA 10 Mk2 (Kruss, Hamburg, Germany). Droplets of ultra-high quality (UHQ) water with a volume of 0.2 μL were placed on the sample surface and contact angles were determined. The presented results were obtained by averaging the results of ten measurements. The microstructure and formation of the apatite layer after incubation in SBF were investigated using a FEI Nova Nano SEM 200 scanning electron microscope (SEM) equipped with an energy dispersive X-ray (EDX) analyzer (EDAX Company, Mahwah, NJ, USA). Samples were coated with carbon before observations. Preliminary assessment of bioactivity was performed according to the procedure described by Kokubo [15]. POM and POM/HAp-g-PCL composites were incubated in a 1.5× concentrated simulated body fluid (SBF) at 37 °C for three weeks. The SBF was changed every three days and the sample mass to SBF volume ratio was 1:10. Next, samples were removed from SBF, rinsed with distilled water, and dried. Dry sample was investigated by using SEM-EDX.

Schiff ’s reagent has been used to detect formaldehyde in filtrates after incubation of POM and POM/HAp-g-PCL in distilled water. Incubation was performed at 37 °C for 3 weeks. The sample mass to water mass ratio was 1:10. The water was changed every 24 h and tests with Schiff’s reagent were performed after 1, 7, and 21 days. In Schiff’s test, the water after incubation is moved to test tubes and three drops of Schiff reagent are added to every test tube. Distilled water was used as a reference sample, and color of filtrates were monitored. According to Schiff’s reagent characteristics, when concentration of formaldehyde is higher than 3 ppm, the color of filtrate is pink.

## 3. Results and Discussion

**Figure 2:**
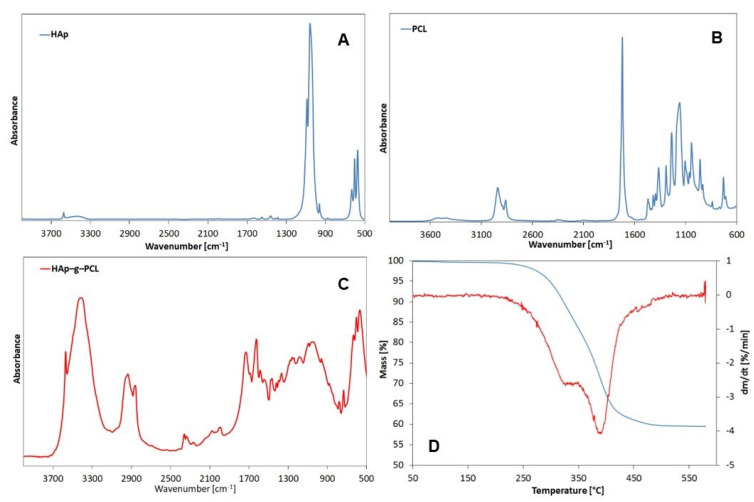
FTIR spectra of HAp (**A**), PCL (**B**) and HAp-g-PCL (**C**) and TGA results of HAp-g-PCL (**D**).

TGA results (Figure 2a) show that the obtained HAp-g-PCL contains 59.86% of HAp and 40.14% of organic phase and is thermally stable at the temperature of POM melt processing. FTIR analysis confirmed chemical grafting of PCL macrochains to HAp (Figure 2b).

**Figure 3:**
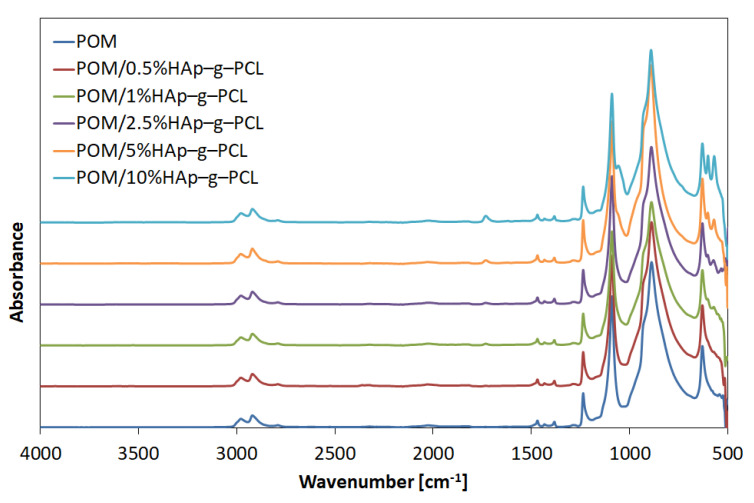
FTIR-ATR spectra of POM and POM/HAp-g-PCL composites.

For HAp, the absorption bands with the highest intensity have been found at 1090, 1057, 962, and 602 cm^−1^, which can be attributed to PO_3_^4−^ motions, while the absorption bands characteristic for the hydroxyl groups in HAp can be found at 633, 602, and 3571 cm^−1^. For HAp-g-PCL, the presence of absorption bands in the range 1740–1680 cm^−1^ from the C=O groups confirmed the formation of urethane bonds during the grafting process [16]. Furthermore, it should be noted that there is a significant decrease in the intensity of absorption bands from stretching vibrations of the OH groups in HAp at 3571 cm^−1^, which confirms the grafting of HAp to HDI. FTIR-ATR spectra of POM/HAp-g-PCL composites are displayed in Figure 3.

The two absorption bands at 2981 and 2923 cm^−1^ can be attributed to symmetric stretching vibrations (ν) of CH_2_ groups, absorption band at 1468 cm^−1^ to bending vibrations (δ) and band at 1381 cm^−1^ to the wagging vibrations of the same groups [17,18]. Strong absorption bands at 1090 cm^−1^ arise from stretching vibrations of C-O-C groups [19,20]. It should be noted that in the FTIR spectra, no changes in band position for any POM/HAp-g-PCL composites were found. Only for composites with a higher content of HAp-g-PCL (5 and 10%) were absorption bands from PO_3_ found at 605 and 565 cm^−1^, respectively. Moreover, for composites with content of HAp-g-PCL higher than 1.0%, absorption bands at 1700–1750 cm^−1^ can be attributed to the PCL carbonyl group or carbonyl group from urethane bond.

**Figure 4:**
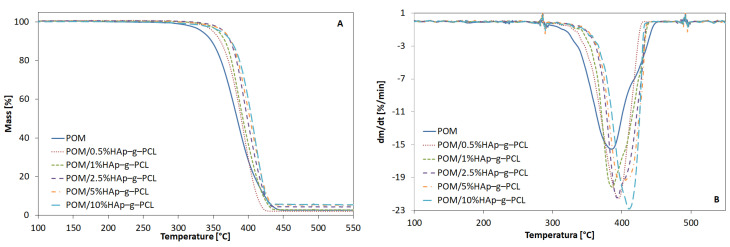
TG (**A**) and DTG (**B**) curves of POM and POM/HAp-g-PCL composites.

Next, the effect of HAp-g-PCL on the thermal stability of the POM matrix has been investigated. The TGA results are presented in Figure 4 and Table 1.

As can be seen from Table 1, the thermal stability of the POM matrix increases with increasing HAp-g-PCL content up to 2.5 wt.% from 292 °C for unmodified POM to 333 °C for the POM/2.5%HAp-g-PCL composites, but for higher HAp-g-PCL contents a decrease in the thermal stability of POM has been observed. In our previous works, a significant decrease in POM thermal stability has been observed after incorporation of higher amounts of nanohydroxyapatite, even up to 30 °C for 10% HAp content in POM copolymers [21]. This effect has been attributed to the catalytic influence of the active basic sites in the structure of HAp which facilitate at higher temperatures POM chain scission and depolymerization to formaldehyde [7]. In this study, basic active sites in HAp (hydroxyl groups) have been used to chemically bond PCL chains using diisocyanate as a coupling agent, and as a result they have been blocked. Furthermore, the presence of a urethane bond with nitrogen atoms can improve the thermal stability of POM; compounds containing nitrogen atoms have been used as a heat stabilizer in POM materials [22]. The combination of these two effects leads to improved POM thermal stability in POM/2.5%HAp-g-PCL composites.

Moreover, some changes can be seen in DTG profiles. For unmodified POM, two maxima have been observed, while with an increase in HAp-g-PCL content the shape gradually changed to one maximum, which suggests changes in the degradation mechanism. Furthermore, an increase in T_DTGmax_ from 386 up to 410 °C has been observed with increasing functionalized HAp content, which also confirms the stabilizing effect of functionalized HAp on the POM thermal stability.

**Figure 5:**
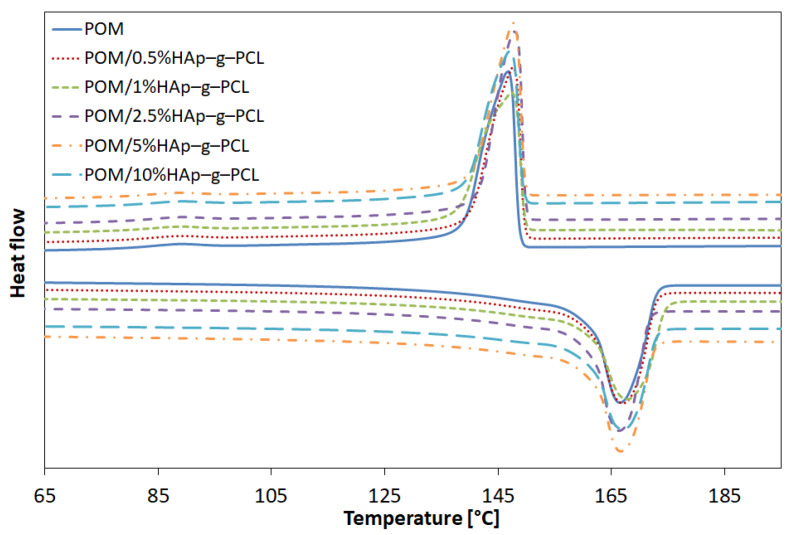
DSC curves for melting (second heating run) and crystallization of POM and POM/HAp-g-PCL composites.

Next, differential scanning calorimetry (DSC) studies have been performed to find the influence of functionalized HAp on POM melting and crystallization processes. On the basis of DSC data, the degree of crystallinity of POM in POM/HAp-g-PCL composites has been calculated using Equation (3):

(3) $$\chi_{c}=\frac{\Delta H-\Delta H_{a}}{\left(1-x\right)\Delta H_{m}^{o}}=\frac{\Delta H_{m}}{\left(1-x\right)\Delta H_{m}^{o}}$$

where $\Delta H_{m}^{o}$ is enthalpy of fusion of 100% crystalline polymer (326.3 J/g for POM [23]), $\Delta H_{m}$ is enthalpy of melting obtained from DSC measurements, and *x* is the mass fraction of HAp-g-PCL in POM/HAp-g-PCL composite [23]. DSC profiles are presented in Figure 5 and Table 2.

In general, for the first heating run, a higher degree of crystallinity has been observed for composites compared to unmodified POM, while for the second heating run the degree of crystallinity was on a similar level, except for the composite with 10% functionalized HAp, where in both heating runs the degree of crystallinity was the highest. These results suggest that functionalized HAp acts as a nucleating agent in the POM crystallization process. A similar effect has been observed in POM/HAp nanocomposites [24]. Moreover, it can be observed that incorporation of functionalized HAp has a negligible impact on the temperatures of both melting and crystallization of POM in POM/HAp-g-PCL composites.

**Figure 6:**
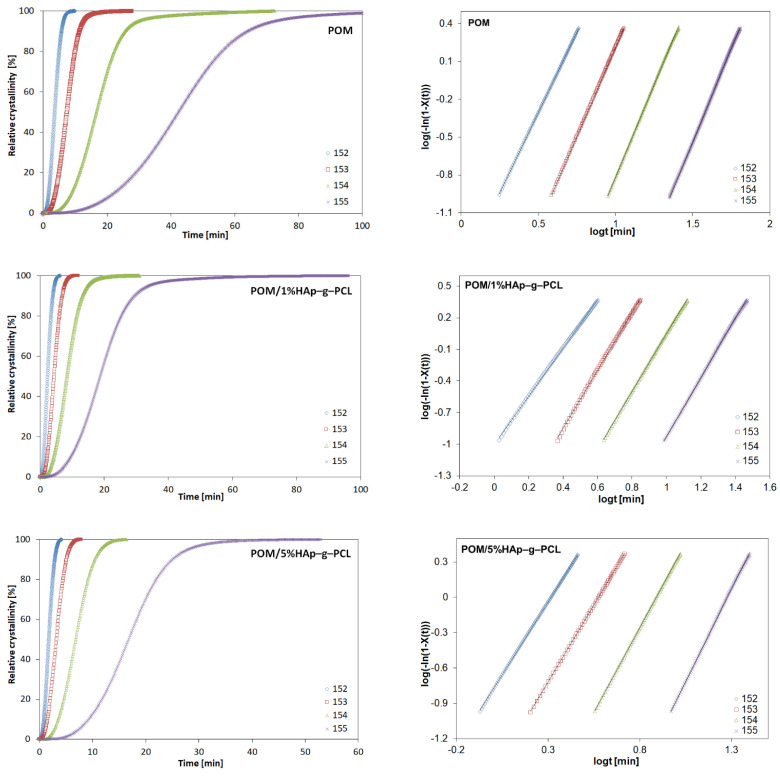
Relative crystallinity vs. time for isothermal crystallization (**left**) and plots of *lg*(−*ln* [1 − *X*(*t*)]) versus *log*(*t*) (**right**) for isothermal crystallization of POM, POM/1%HAp-g-PCL and POM/5%HAp-g-PCl.

To determine the exact course of the crystallization process and to determine its nature during cooling, a study of the crystallization kinetics was carried out. The first three graphs show the curves of relative crystallinity versus time for three selected composites that crystallized isothermally at certain temperatures (Figure 6).

An increase in relative crystallinity as a function of time can be observed, as well as the effect of the crystallization temperature on the rate of crystallization. In the first stage, with the relative crystallinity values up to 85%, a rapid increase in crystallinity (primary crystallization) was observed; in the next stage, the crystallization slows down—secondary crystallization, associated with recrystallization processes, occurs. A similar effect was observed for the POM/HAp composites [25]. The Avrami equation [25] was applied to calculate the crystallization kinetics:

(4) $$X_{t}=1-\exp\left(-kt^{n}\right)$$

where: $X_{t}$ is the relative crystallinity as a function of time *t*, *k* is the Avrami rate constant, and *n* the Avrami exponent. It describes the nucleation mechanism (heterogeneous or homogeneous) and the dimensionality of the growing crystals. This equation can be converted to logarithmic form:

(5) $$log\left[-ln\left(1-X_{t}\right)\right]=logk+nlogt$$

**Figure 7:**
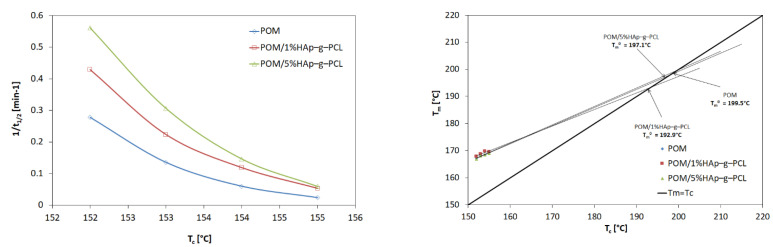
Overall crystallization rate expressed as the inverse of half crystallization time (*t*_1/2_) vs. isothermal crystallization temperature (*Tc*) (**left**) and Hoffman–Weeks plot for POM and POM/HAp-g-PCL composites (**right**).

Using this equation, appropriate graphs (Figure 7) were constructed in the range of 10–90% relative crystallinity and *n* and *logk* were determined, see Table 3.

A linear dependence was observed in the range of 10–90% relative crystallinity (*R*^2^ ~ 0.9–1). The crystallization half-time, *t*_1/2_, defined as the time at which the degree of crystallization reaches 50%, is determined from the following relationship.

(6) t12=(ln2k)1/n 

The crystallization rate (*G*) was calculated as the reciprocal of *t*_1/2_ [26]. In the next step, the time taken for the system to reach its maximum crystallization rate t_max_ was calculated [27]:

(7) tmax=(n−1nk)1n

Results presented in Table 4 shows that the values of the Avrami exponent are in the range of 2.28 to 3.15. This suggests two-dimensional and/or three-dimensional growth of the crystals. For pure POM, these values are 2.56–2.97, which can be attributed to the growth of three-dimensional (spherulitic) crystals.

A slight increase in the calculated Avrami exponent in this case suggests that HAp-g-PCL acts as a nucleating agent and thus increases the crystallinity of the composite samples. Additionally, the overall rate of crystallization decreases with increasing temperature. This is due to the fact that the kinetics of crystallization is strongly influenced by the nucleation process close to the temperature *T_m_*. The highest values of the crystallization rate are achieved for the composite POM/5%HAp-g-PCL, and an increase in the crystallization rate is observed with increasing HAp-g-PCL content. For POM/HAp composites, a decrease in the overall rate of crystallization was observed with an increasing additive content, which was attributed to the hindering chain movement effect in the presence of the nanometric size modifier [28].

The next step was to determine the equilibrium melting temperature (i.e., the melting point of the infinitely high average molecular weight polymer) by linear extrapolation of the data from the relationship *T_m_* vs. *T_c_*, i.e., the value of the melting point obtained for samples after isothermal crystallization at selected crystallization temperatures (Hoffman–Weeks plot), see Figure 7.

For composites containing HAp-g-PCL, *T_m_*^0^ decreased, indicating that during crystallization, more defective spherulite (or lamellar) crystals were formed. The large number of nuclei formed as a result of the nucleation on the HAp-g-PCL surface resulted in a large number of spherulites being formed in a limited (congested) space, which hampers their growth and facilities defects formation. The temperature drop may also suggest that the crystalline phase of composites is more defected than that of pure POM.

**Figure 8:**
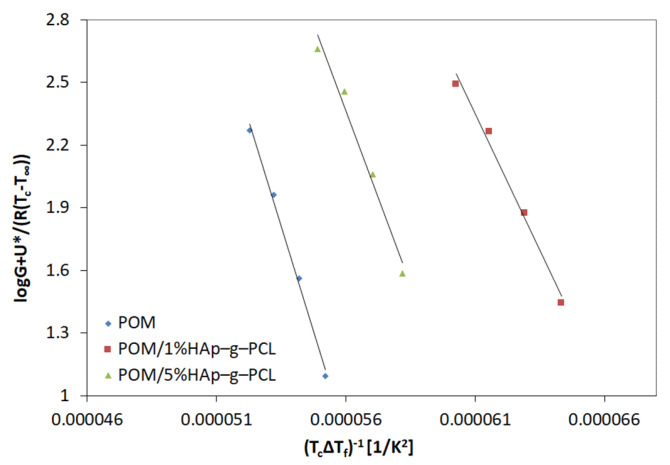
Plot of *logG* + [U*/*R*(*T_c_* − *T_∞_*)] vs. 1/(*T_c_*∆*T_f_*) for POM, POM/1% and POM/5% HAp-g-PCL.

The plots of *logG* + [U*/*R*(*T_c_*−*T_∞_*)] vs. *1/(T_c_*∆*T_f_)* for POM and POM in POM/HAp-g-PCL composites are collected in Figure 8.

The G value was assumed as the reciprocal of the half-crystallization time, U* is the activation energy of the molecular transfer taking a constant value of 6.3 kJ/mol, *R* is the gas constant of 8.314 J/mol·K, *T_c_* is the crystallization temperature, and *T* is taken as the hypothetical temperature 30 K lower than the polymer glass transition temperature *T_g_*. *K_g_* is the energy barrier and depends on the degree of supercooling [29] and can be determined from the linear plot of *logG* + [U*/*R*(*Tc* − *T∞*)] plotted against (*Tc*Δ*Tf*)^−1^.

By assuming the regime III of crystallization, the $\sigma_{e}$ value can be estimated from equation:

(8) $$K_{g}=\frac{nb\sigma \sigma_{e}T_{m}^{0}}{\Delta h_{f}^{0}k_{b}}$$

where *n* takes the value 4 for crystallization regimes I and III, b is the thickness of the surface nucleus and has a value of 4.46 × 10^−10^ m, σ is the lateral surface free energy, $\Delta h_{f}^{0}$ is the heat of fusion per unit volume of crystal (3.55 × 10^8^ J·m^−3^), a *k_B_* is the Boltzmann constant.

The thickness of the lamellas (l) was calculated on the basis of the Gibbs–Thomson equation [30].

(9) $$T_{m}=T_{m}^{0}\left(1-\frac{2\sigma_{e}}{\Delta h_{o}l}\right)$$

where $\Delta h_{o}$ is thermodynamic enthalpy of fusion per unit volume of the crystalline phase. The results obtained have been collected in Table 4.

The results obtained show that the introduced HAp-g-PCL lowers the energy barrier of the crystallization process and thus can be considered as an effective nucleant for the POM crystallization process.

Analysis of the kinetics of the crystallization of POM/HAp-g-PCL composites showed that the introduction of HAp-g-PCL causes a heterogeneous nucleation and the formation of more perfect crystals, as evidenced by an increase in the Avrami exponent (*n*) and leads to formation crystals with higher lamellar thickness. As for the half-crystallization time and the nucleation energy barrier, a decrease in these values was observed.

**Figure 9:**
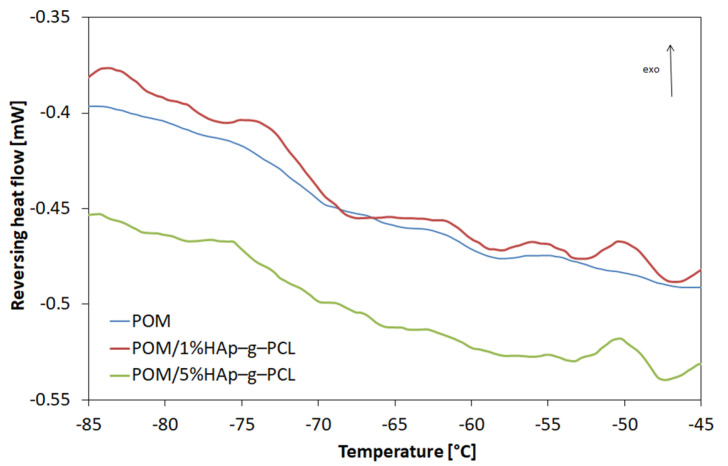
TOPEM DSC curves (reversing heat flow) for POM and POM/HAp-g-PCL.

To analyze the melting process of composites in more detail and to determine the glass transition temperature, the DSC TOPEM study, i.e., differential scanning calorimetry with temperature modulation, was carried out. TOPEM DSC reversing heat flow profiles are presented in Figure 9.

Based on this study, the glass transition temperatures and changes in specific heat in the area of the glass transition were determined, which are listed in Table 5.

It was found that as the modifier content increases, the glass transition temperature decreases, and for 5% HAp-g-PCL content it reaches the lowest value. This may suggest that HAp-g-PCL can exert a plasticization action.

**Figure 10:**
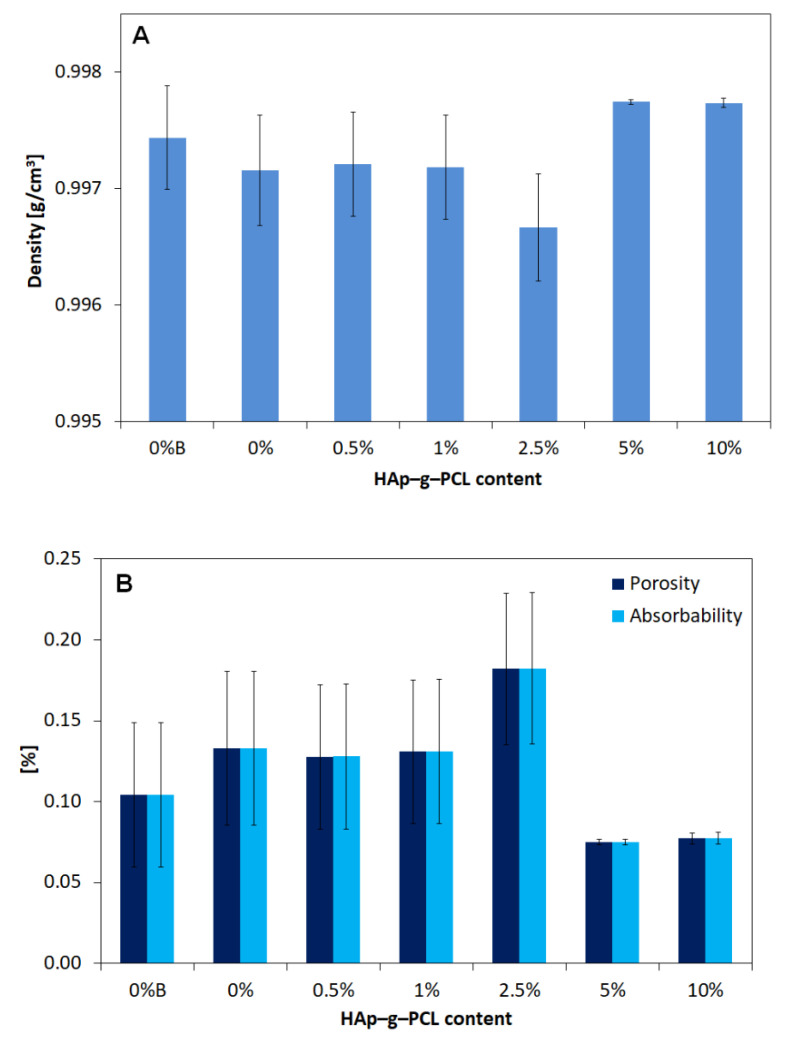
Apparent density (**A**) and moisture absorption (**B**) for POM and POM/HAp-g-PCL composites.

Next, based on the masses of the tested samples determined by hydrostatic weighing, the apparent density (*D_p_*), porosity (*P_o_*), and water absorption (*N_w_*) were calculated (Figure 10).

**Figure 11:**
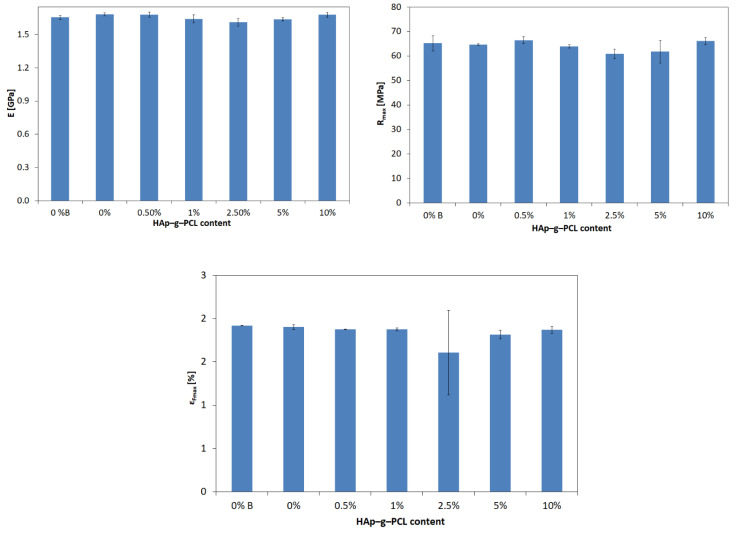
Young’s modulus determined from the graphs *σ* = *f* (ɛ) obtained in the uniaxial tensile test.

After analysis of the results presented in Figure 10, it can be seen that all composite samples are characterized by a very similar density, differing from the extruded POM sample by 0.003–0.058% (0.03–0.58 $\frac{\mathrm{kg}}{m^{3}}$). In most cases, the modifying phase slightly increases the density compared to the reference sample. The highest density and the lowest standard deviation were observed for samples containing the highest percentages of reinforcement: 5% (D_p_ = 997.75 $\frac{\mathrm{kg}}{m^{3}}$) and 10% (997.74 $\frac{\mathrm{kg}}{m^{3}})$. However, the lowest density (D_p_ = 996.67 $\frac{\mathrm{kg}}{m^{3}}$) was obtained for a sample containing 2.5% HAp-g-PCL. Moreover, the POM sample not subjected to extrusion is characterized by a slightly higher density (by 0.03% − 0.28$\frac{\mathrm{kg}}{m^{3}}$) than the processed polymer. This effect can be connected with incorporation of additional pores in the material during the extrusion process (as evidenced by the porosity results described below). The more pores a material has, the more water it can absorb. The results of water absorption and porosity for individual tested fittings are almost equal; the differences occur in parts smaller than hundredths (for this reason, they are presented in one diagram). In most cases, the addition of HAp-g-PCL showed a decrease in porosity compared to that of the reference sample. The composite shapes tested achieved an average porosity/water absorption at a similar level. Moreover, the obtained results confirm the dependence that the higher the density, The lower the porosity. Next, the tensile strength has been investigated; the results are presented in Figure 11.

Tensile strength was determined from three measurements for each composition. The results obtained revealed that the incorporation of HAp-g-PCL up to 10% does not significantly affect the tensile strength (*R_m_*). The results obtained for composite samples differ from those for the reference sample (0%) within 1.3–5.9% (0.8–3.8 Mpa).

Among the tested compositions, POM/0.5% HAp-g-PCL has the highest *R_m_*. Interestingly, a slightly lower tensile strength (only by 0.005%) was recorded for the POM/10% HAp-g-PCL composite. On the other hand, the lowest value of this parameter was found for POM/2.5% HAp-g-PCL. It can be concluded that the addition of HAp-g-PCL to the POM matrix for most of the tested samples reduces the elasticity of the material. The tested polymer (0%) achieved slightly higher elongation at maximum force (*ε_Fmax_* = 5.00 mm) by 1.5–4.7% (0.07–0.78 mm) than composite samples. All tested composites were characterized by a similar Young’s modulus, differing from each other in the range of 0.2–4.3% (3.5–71.7 Mpa). Therefore, it can be concluded that the addition of the HAp-g-PCL has a small impact on the Young’s modulus.

**Figure 12:**
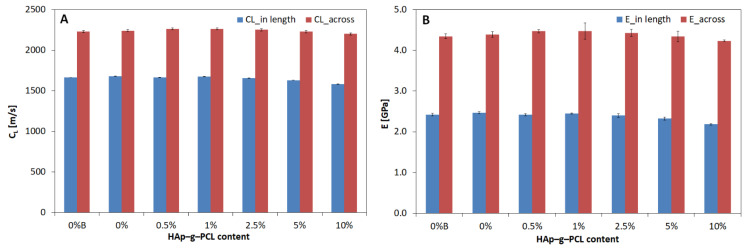
Ultrasonic wave velocity (**A**) and Young’s modulus (*E*) (**B**) determined by the shadow transmission method for POM-HAp-g-PCL, *C_L_*_1_ and *E*_1_ samples—wave direction: along the sample length, *C_L_*_2_ and *E*_2_ wave direction along the paddle width.

An ultrasonic wave with a frequency of 1 MHz was passed along the length and width of the obtained composites, and its velocity was determined as the average of three measurements. On the basis of the obtained wave velocities (*C_L_*), Young’s modulus (*E*) was calculated. The distribution of *C_L_* and *E* values is shown in Figure 12.

The tests performed show that the velocity of the ultrasonic wave and the modulus of elasticity decrease with increasing HAp-g-PCL content (up to 10%) in the POM matrix. Composite samples containing up to 2.5% HAp-g-PCL have much lower Young’s modulus values than extruded POM and samples containing 5% to 10% HAp-g-PCL. The highest wave propagation velocity was recorded for the extruded POM sample and the lowest for the sample with the highest content of HAp-g-PCL. All composite materials tested by means of an ultrasonic wave (running along the width of the fittings) are characterized by a similar speed of propagation (*C_L_*_2_). When analyzing the obtained results, it can be concluded that the percentage of HAp-g-PCL has little effect on the value of the properties discussed. The addition of HAp-g-PCL in amounts up to 2.5% increased and in amounts from 5% to 10% decreased the Young’s modulus of the tested composites. The composite containing 1% HAp-g-PCL was characterized by the highest *E* of 4471.7 MPa. Moreover, it can be concluded that the Young’s modulus determined by means of an ultrasonic wave conducted along the width of polyoxymethylene samples increased slightly by 0.9% (41.4 MPa) as a result of the extrusion technology features (similar to the Young’s modulus determined by means of an ultrasonic wave guided along the length of the fittings).

**Figure 13:**
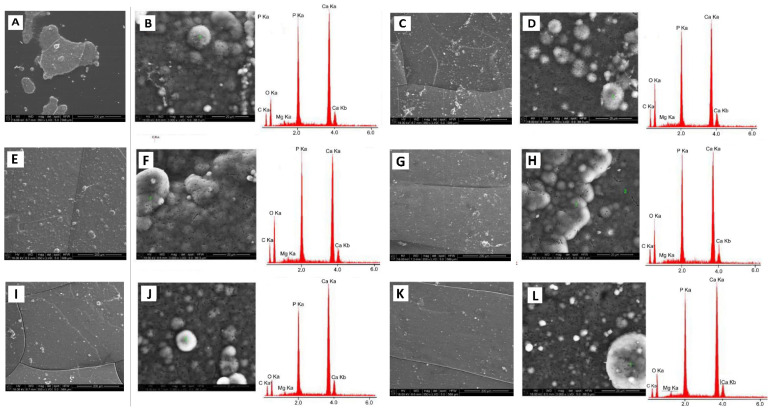
SEM-EDX results for POM and POM/HAp-g-PCL composites after 21 days of incubation in SBF: (**A**,**B**) POM, (**C**,**D**) POM/0.5% HAp-g-PCL, (**E**,**F**) POM/1.0% HAp-g-PCL, (**G**,**H**) POM/2.5% HAp-g-PCL, (**I**,**J**) POM/5% HAp-g-PCL, and (**K**,**L**) POM/10% HAp-g-PCL.

The preliminary bioactivity assessment was then performed using the Kokubo method [15]. The SEM-EDX results for samples incubated in simulated body fluid (SBF) are presented in Figure 13.

Based on SEM-EDX results, one can see that after incubation in SBF, a layer of apatite was formed on the surface of all tested materials. This may evidence that both POM and POM/HAp-g-PCL composites are bioactive materials. Moreover, it was observed that the addition of HAp-g-PCL increases the bioactivity of the material compared to that of unmodified POM. For unmodified POM, a discontinuous and nonuniform layer of apatites has been observed on the sample surface, while for samples modified with HAp-g-PCL a continuous, more dense apatite layer occurred, as can be seen in Figure 13. EDX analysis performed at selected points showed that the calculated Ca/P molar ratio for POM, POM/2.5%HAp-g-PCL, POM/5%HAp-g-PCL, and POM/10%HAp-g-PCL was 1.52, 1.50, 1.52, and 1.54, respectively, indicating the presence of a nonstoichiometric hydroxyapatite with calcium deficiency. There is also a minor amount of nonstoichiometric HAp with excess of calcium—Ca/P molar ratio greater than 1.667 [31]. Deviations from stoichiometry are the result of the presence of various isomorphic substitutions in the crystal structure of HAp; in the case of the samples analyzed, these are substitutions in the form of Mg^2+^ ions. Their presence was recorded in the EDS analysis as they were also present in SBF.

**Figure 14:**
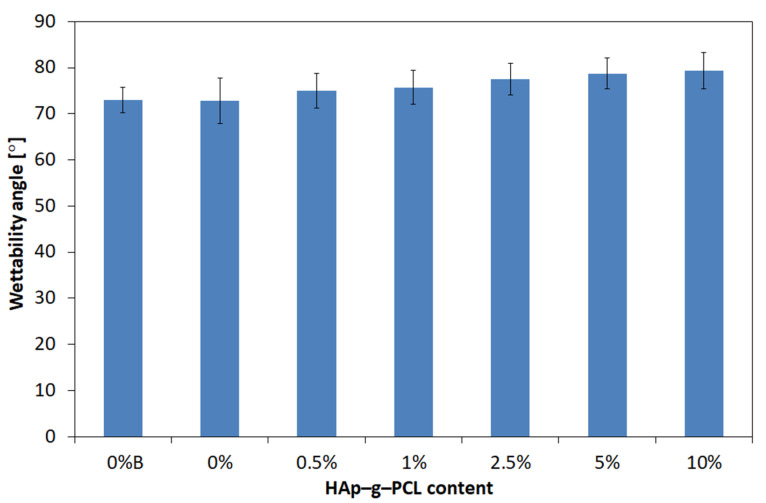
Wettability of POM and POM/HAp-g-PCL composites.

Wettability tests (Figure 14) showed that the surfaces of all tested samples were hydrophilic with a contact angle below 90°.

It was also observed that the contact angle increased slightly with an increase in the amount of HAp-g-PCL in the composite. The highest wetting angle, 79.3°, was recorded for the sample containing the highest amount of HAp-g-PCL, and the lowest value of 72.9° was obtained for unmodified POM.

**Figure 15:**
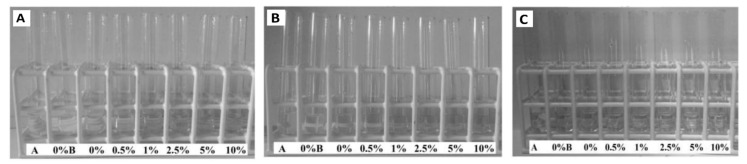
Colorimetric assay of formaldehyde release from POM/HAp-g-PCL composites after 1 (**A**), 7 (**B**), and 21 (**C**) days.

The next step was to investigate the release of formaldehyde from POM and POM/HAp-g-PCL composites during incubation in distilled water at 37 °C. As POM thermal degradation proceeds mostly through a depolymerization mechanism, with formaldehyde being the main volatile product released, determination of its concentration is of primary importance for any biomedical application. Hence, the presence of formaldehyde in the filtrates after incubation was tested using Schiff’s base. The tests carried out showed that formaldehyde is released from the tested samples in amounts acceptable for biomedical applications, less than 3ppm, even in materials containing large amounts of HAp-g-PCL, see Figure 15.

Previous studies on the POM/HAp composites showed that the thermal stability of POM composites decreased significantly as a result of incorporation of HAp up to 10% by weight, leading to an excessive release of formaldehyde from composites under incubation conditions [32]. This detrimental effect was not observed for POM/HAp-g-PCL composite materials studied in the course of this work, which may open new perspectives for new applications in, e.g., orthopedic areas.

## 4. Conclusions

We have designed and developed new composite materials based on polyoxymethylene suitable for medical applications such as, e.g., long-term bone implants. The results of thermal tests on composites with different contents of HAp-g-PCL hybrid modifier confirmed the increase in the thermal stability of POM by more than 40 °C, in contrast to the POM/HAp composites tested earlier, where the introduction of HAp caused a significant deterioration of the thermal stability of polymer matrix. FTIR tests confirmed the reduction in the number of -OH groups, which resulted in an increase in the thermal stability of POM on the HAp surface and the presence of urethane bonds formed by HDI linker used to graft PCL on HAp surface. The results of DSC, DSC TOPEM, and isothermal crystallization kinetics confirmed that the introduced hybrid additive acts as a nucleant in the POM crystallization process, leading to an increase in the crystallinity of the samples, but only to the content of 1–2.5% HAp-g-PCL. The results of kinetic calculations indicate crystallization of POM with the formation of spherulitic structures. All composite samples obtained were characterized by a very similar density. Furthermore, the results obtained revealed that incorporation of HAp-g-PCL up to 10% does not significantly affect tensile strength (*R_m_*). Wettability tests revealed that the contact angle increased slightly with an increasing amount of HAp-g-PCL in the composite. It was also confirmed that formaldehyde is released from the tested samples in an amount acceptable in biomedical applications, less than 3 ppm, even in composites containing large amounts of HAp-g-PCL.

## Figures and Tables

**Table 1:** Thermal stability parameters for POM and POM/HAp-g-PCL composites.

Material	T_1%_ (°C)	T_3%_ (°C)	T_5%_ (°C)	T_10%_ (°C)	T_25%_ (°C)	T_50%_ (°C)	T_DTGmax_ (°C)
POM	292	341	349	359	375	390	386
POM/0.5%HAp-g-PCL	326	348	356	366	379	392	395
POM/1%HAp-g-PCL	328	348	356	366	379	392	386
POM/2.5%HAp-g-PCL	333	355	364	374	386	398	393
POM/5%HAp-g-PCL	325	354	364	375	388	402	405
POM/10%HAp-g-PCL	312	348	361	375	391	405	410

**Table 2:** Temperatures, heat of phase transitions, and degree of crystallinity for POM and POM/HAp-g-PCL composites.

Sample	T_onset_ (°C)	T_endset_ (°C)	T_max_ (°C)	Heat of Phase Transition (J/g)	Degree of Crystallinity (%)
First heating run					
POM	159	167	171	164	44.3
POM/0.5%HAp-g-PCL	160	168	171	144	47.4
POM/1%HAp-g-PCL	158	172	176	153	48.5
POM/2.5%HAp-g-PCL	158	169	174	156	46.8
POM/5%HAp-g-PCL	159	168	173	146	45.6
POM/10%HAp-g-PCL	158	168	171	137	48.8
Crystallization					
POM	149	147	139	167	
POM/0.5%HAp-g-PCL	150	148	140	169	
POM/1%HAp-g-PCL	150	147	138	167	
POM/2.5%HAp-g-PCL	150	148	141	162	
POM/5%HAp-g-PCL	150	148	140	156	
POM/10%HAp-g-PCL	150	147	139	150	
Second heating run					
POM	160	167	173	169	51.8
POM/0.5%HAp-g-PCL	160	168	173	163	50.4
POM/1%HAp-g-PCL	160	169	174	163	50.9
POM/2.5%HAp-g-PCL	161	167	173	162,	51.8
POM/5%HAp-g-PCL	161	168	172	153	51.0
POM/10%HAp-g-PCL	160	168	173	152	55.0

**Table 3:** Avrami exponent (*n*) and *k* for crystallization of POM in POM/HAp-g-PCL composites.

Sample	T_C_ (°C)	*n*	*k* (min^−*n*^)	*R* ^2^	*t_max_* (min)	*t*_1/2_ (min)	G (min^−1^)
**POM**	152	2.56	2.62 × 10^−2^	1.0000	3.42	3.60	0.2778
	153	2.84	2.38 × 10^−3^	1.0000	7.22	7.39	0.1353
	154	2.94	1.75 × 10^−4^	0.9997	16.45	16.73	0.0598
	155	2.97	1.01 × 10^−5^	0.9998	41.73	42.34	0.0236
**POM/1%HAp-g-PCL**	152	2.28	1.00 × 10^−1^	0.9995	2.13	2.33	0.4287
	153	2.75	1.13 × 10^−2^	0.9993	4.34	4.47	0.2235
	154	2.74	2.02 × 10^−3^	0.9998	8.15	8.42	0.1188
	155	2.80	1.86 × 10^−4^	0.9996	18.30	18.80	0.0532
**POM/5%HAp-g-PCL**	152	2.47	1.66 × 10^−1^	0.9999	1.68	1.78	0.5606
	153	2.62	3.12 × 10^−2^	1.0000	3.13	3.27	0.3062
	154	2.87	2.78 × 10^−3^	1.0000	6.69	6.84	0.1463
	155	3.15	9.57 × 10^−5^	0.9998	16.76	16.84	0.0594

**Table 4:** *K_g_* and *σ_e_* parameters for POM and POM/HAp-g-PCL.

Sample	*K_g_* × 10^5^ (K^2^)	*T_m_*^0^ (K)	U* (kJ/mol)	$\sigma_{e}\;(J/m^{2})$	l (nm) (dla 152 °C)
POM	4.03	199.5	6.3	0.159	17.9
POM/1%HAp-g-PCL	3.32	192.9	6.3	0.133	21.1
POM/5%HAp-g-PCL	2.60	197.1	6.3	0.103	27.4

**Table 5:** Glass temperature and ∆C_p_ for POM and POM/HAp-g-PCL composites (based on TOPEM DSC results, determined from reversing heat flow).

Material	Glass Temperature (°C)	∆C_p_ (J/g·K)
POM	−71.2	0.068
POM/1%HAp-g-PCL	−71.2	0.110
POM/5%HAp-g-PCL	−73.9	0.097

## Data Availability

Not applicable.

## References

[1] Lüftl S., Visakh P.M., Chandran S. (2014). Polyoxymethylene Handbook: Structure, Properties, Applications and their Nanocomposites.

[2] Hu Y., Ye L. (2006). Study on the Thermal Stabilization Effect of Polyamide on Polyoxymethylene. Polym. Plast. Technol. Eng..

[3] Pielichowska K. (2012). Polyoxymethylene-Homopolymer/Hydroxyapatite Nanocomposites for Biomedical Applications. J. Appl. Polym. Sci..

[4] Moore D.J., Freeman M.A.R., Revell P.A., Bradley G.W., Tuke M. (1998). Can a total knee replacement prosthesis be made entirely of polymers?. J. Arthroplast..

[5] Meyers M.A., Chen P.-Y., Lin A.Y.-M., Seki Y. (2008). Biological materials: Structure and mechanical properties. Prog. Mater. Sci..

[6] Pielichowska K., Blazewicz S. (2010). Bioactive polymer/hydroxyapatite (nano)composites for bone tissue regeneration. Adv. Polym. Sci..

[7] Pielichowska K. (2015). Thermooxidative degradation of polyoxymethylene homo- and copolymer nanocomposites with hydroxyapatite: Kinetic and thermoanalytical study. Thermochim. Acta.

[8] Pielichowska K., Król K., Majka T.M. (2016). Polyoxymethylene-copolymer based composites with PEG-grafted hydroxyapatite with improved thermal stability. Thermochim. Acta.

[9] Król-Morkisz K., Karaś E., Majka T.M., Pielichowski K., Pielichowska K. (2019). Thermal Stabilization of Polyoxymethylene by PEG-Functionalized Hydroxyapatite: Examining the Effects of Reduced Formaldehyde Release and Enhanced Bioactivity. Adv. Polym. Technol..

[10] White T., Ferraris C., Kim J., Madhavi S. (2005). Apatite—An Adaptive Framework Structure. Rev. Mineral. Geochem..

[11] Kreidler E.R., Hummel F.A. (1970). The crystal chemistry of apatite: Structure fields of fluor- and chlorapatite. Am. Mineral..

[12] Joris S.J., Amberg C.H. (1971). Nature of deficiency in nonstoichiometric hydroxyapatites. I. Catalytic activity of calcium and strontium hydroxyapatites. J. Phys. Chem..

[13] Archodoulaki V.M., Luftl S., Seidler S. (2008). Stabiliser consumption of polyoxymethylene investigated by means of the pressure oxidative induction time method. Polym. Test..

[14] Krautkrämer J., Krautkrämer H. (1990). Introduction. Ultrasonic Testing of Materials.

[15] Kokubo T., Takadama H. (2006). How useful is SBF in predicting in vivo bone bioactivity?. Biomaterials.

[16] Król P. (2007). Synthesis methods, chemical structures and phase structures of linear polyurethanes. Properties and applications of linear polyurethanes in polyurethane elastomers, copolymers and ionomers. Prog. Mater. Sci..

[17] Ma Z.-L., Tsou C.-H., Yao Y.-L., de Guzman M.R., Wu C.-S., Gao C., Yang T., Chen Z.-J., Zeng R., Yang T.-T. (2021). Thermal Properties and Barrier Performance of Antibacterial High-Density Polyethylene Reinforced with Carboxyl Graphene-Grafted Modified High-Density Polyethylene. Ind. Eng. Chem. Res..

[18] Tsou C.-H., Yao W.-H., Wu C.-S., Tsou C.-Y., Hung W.-S., Chen J.-C., Guo J., Yuan S., Wen E., Wang R.-Y. (2019). Preparation and characterization of renewable composites fromPolylactide and Rice husk for 3D printing applications. J. Polym. Res..

[19] Rahman M.D.S., Shaislamov U., Yang J.-K., Kim J.-K., Yu Y.H., Choi S., Lee H.J. (2016). Effects of electron beam irradiation on tribological and physico-chemical properties of Polyoxymethylene copolymer (POM-C). Nucl. Instrum. Methods Phys. Res. Sect. B Beam Interact. Mater. At..

[20] Wen Y., Tsou C.-H., Gao C., Chen J.-C., Tang Z., Chen Z., Yang T., Du J., Yu Y., Suen M.-C. (2020). Evaluating distillers grains as bio-fillers for high-density polyethylene. J. Polym. Res..

[21] Pielichowska K., Szczygielska A., Spasówka E. (2012). Preparation and characterization of polyoxymethylene-copolymer/hydroxyapatite nanocomposites for long-term bone implants. Polym. Adv. Technol..

[22] Fukuda H., Ishida S., Matsuoka T. (1975). Polyacetal Composition. U.S. Patent.

[23] Wunderlich B. (2005). Thermal Analysis of Polymeric Materials.

[24] Pielichowska K., Dryzek E., Olejniczak Z., Pamula E., Pagacz J. (2013). A study on the melting and crystallization of polyoxymethylene-copolymer/hydroxyapatite nanocomposites. Polym. Adv. Technol..

[25] Avrami M. (1941). Granulation, Phase Change, and Microstructure Kinetics of Phase Change. III. J. Chem. Phys..

[26] Chatterjee T., Lorenzo A.T., Krishnamoorti R. (2011). Poly(ethylene oxide) crystallization in single walled carbon nanotube based nanocomposites: Kinetics and structural consequences. Polymer.

[27] Lin C.C. (1983). The rate of crystallization of poly(ethylene terephthalate) by differential scanning calorimetry. Polym. Eng. Sci..

[28] Müller A.J., Arnal M.L., Trujillo M., Lorenzo A.T. (2011). Super-nucleation in nanocomposites and confinement effects on the crystallizable components within block copolymers, miktoarm star copolymers and nanocomposites. Eur. Polym. J..

[29] Hoffman J.D., Lauritzen J.I. (1961). Crystallization of Bulk Polymers with Chain Folding: Theory of Growth of Lamellar Spherulites. J. Res. Natl. Bur. Stand. Sect. A Phys. Chem..

[30] Wunderlich B. (2013). Macromolecular Physics: Crystal Melting.

[31] Nakamura S., Yamashita K. (2000). Non-Stoichiometric Hydroxyapatite Microcrystals Formation. High Ph Reg. Phosphorus Res. Bull..

[32] Pielichowska K. (2012). The influence of molecular weight on the properties of polyacetal/hydroxyapatite nanocomposites. Part 2. In vitro assessment. J. Polym. Res..

